# The Landmark Series: Therapeutic Cancer Vaccine Strategies for Cold Tumors

**DOI:** 10.1245/s10434-025-17281-1

**Published:** 2025-05-05

**Authors:** Alex B. Blair, Lei Zheng, Kevin C. Soares

**Affiliations:** 1 Division of Surgical Oncology, Department of Surgery, The Ohio State University Wexner Medical Center, Columbus, OH USA; 2 Mays Cancer Center at the University of Texas Health San Antonio MD Anderson Cancer Center, San Antonio, TX USA; 3 Department of Oncology and Department of Surgery, Johns Hopkins University School of Medicine, Baltimore, MD USA; 4 Hepatopancreatobiliary Service, Department of Surgery, Memorial Sloan Kettering Cancer Center, New York, NY USA; 5 David M. Rubenstein Center for Pancreatic Cancer Research, Memorial Sloan Kettering Cancer Center, New York, NY USA

## Abstract

Immunologically cold tumors present a significant challenge in cancer treatment due to their limited baseline immune infiltration and resistance to immunotherapy. Cancer vaccines offer a promising strategy to overcome this barrier by introducing high-quality, tumor-relevant antigens that can stimulate an effective anti-tumor immune response. Therapeutic cancer vaccines are being explored in the neoadjuvant, adjuvant, and minimal residual disease contexts to enhance immune activation and promote immune cell infiltration and function, with the goal to eradicate malignant cells and improve patient survival. Critical hurdles remain in optimizing antigen selection, determining the most effective vaccine formulations, and defining the ideal clinical setting for vaccine use. Moreover, rational combinations of cancer vaccines with other immune modulators (e.g., adjuvants, immune checkpoint inhibitors, and cytokines) may hold the key to enhancing vaccine efficacy and expanding therapeutic options for difficult-to-treat malignancies. This review examines current advancements in cancer vaccines and their utilization for immunologically cold tumors in the perioperative setting, highlighting ongoing challenges and future directions in this evolving field.

Dating back to 1891, Dr. William Coley observed therapeutic effects in sarcoma patients repeatedly inoculated with *Streptococcus pyogenes* and *Seratia marcescens*.^1,2^ In 1962, a controlled study validated the efficacy of Coley’s approach, documenting a 20% treatment response rate following injection of the toxin.^3^ In many ways, this was the first “therapeutic cancer vaccine,” aimed at eliminating active cancer cells by eliciting immune system recognition. While cancer vaccination strategies have evolved beyond the injection of small amounts of pathogen, modern approaches have been met with mixed success. This review seeks to explore the current landscape of cancer vaccine strategies with a focus on their use in cold immunosuppressive solid tumors.

## Immunotherapy for the Immunologically Cold Tumor

**Fig. 1:**
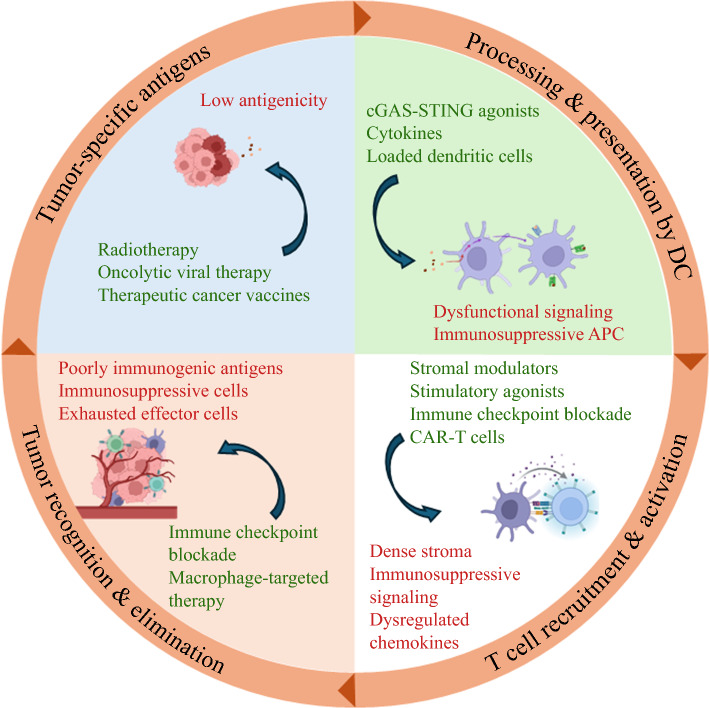
Multi-step interactions in anti-tumor immunity and the immunosuppressive tumor microenvironment: implications for rational combination immunotherapy

The immune response to cancer occurs in a series of regulated steps, shaped by cells and pathways that prevent unchecked inflammation and autoimmunity (Fig. 1). Clinically relevant cancers have, by definition, developed mechanisms to evade immune system recognition and prevent effective anti-tumor immune cell activation.

A cancer vaccine interrupts the immunosuppressive defenses of cancer, permitting effective presentation of antigens, which leads to effective anti-tumor function. A subset of tumors such as melanoma, renal cell carcinoma, and non-small cell lung cancer (NSCLC) are considered “hot” due to their high baseline immune cell infiltration and elevated tumor mutational burden (TMB), providing an abundance of tumor neoantigens. This creates opportunity for a broad armamentarium of immune-based therapeutic strategies including cancer vaccines, immune checkpoint inhibitors, immune activators, and monoclonal antibodies. These approaches aim to reinvigorate a preexisting anti-tumor immunity by shifting the balance from immunosuppression to infiltration by immune effectors. A comprehensive review of cancer vaccines for hot tumors, including melanoma and NSCLC, was recently published in a parallel Landmark Series.^4^

Conversely, tumors such as pancreatic adenocarcinoma (PDAC), triple-negative breast cancer (TNBC), and hepatocellular carcinoma (HCC) are widely considered to be immunologically “cold.” These tumors, at baseline, have a low TMB (hence limited neoantigens) and an immunosuppressive tumor microenvironment (TME) preferentially infiltrated with regulatory cells that impede an effective immune response.^5^ For these cancer subtypes, therefore, current immunotherapeutic approaches are less successful, requiring alternative tactics for antigen identification, presentation, and immune activation.

## Tumor Antigens

A vaccine’s efficacy is dependent on the identification of tumor antigens that are stable, consistently expressed across tumor cell clones, and stimulate an immune response. Tumor antigens can be broadly split into tumor-associated antigens (TAAs) and tumor-specific antigens (TSAs). The TAAs are proteins, including mutated oncoproteins, overexpressed proteins, tissue differentiation antigens, and cancer germline antigens, which are present in normal tissues but abnormally expressed, overexpressed, or modified in cancer cells.

The TSAs, alternatively, arise from mutations or unique post-translational modifications that generate novel epitopes, leading to exclusive expression in tumor cells and absence in normal tissues. Neoantigens are TSAs with mutations that have escaped central tolerance and can be recognized as non-self but have no shared epitopes with other patients. Whereas canonincal neoantigens arise from mutations in coding regions of the genome, non-canonical neoantigens arise from unconventional sources such as alternative splicing, modifications to an RNA transcript, or non-coding mutations. These present unique challenges for effective neoantigen prediction. Additionally, more immunogenic cancer cell clones are eliminated by the immune system, leading to a bottleneck predominance of less immunogenic cell cohorts. Thus, in addition to newly generated mutations, neoantigens can evolve over time, confounding the prediction of quality neoantigens.^6^

Various tools have been developed to predict peptide-major histocompatibility complex (MHC)-binding affinity, including for non-canonical sequences. Neoantigens with high immunogenic potential are more likely to activate T cell responses, enhancing immune memory against the tumor and potentially prolonging patient survival.^6–8^

Several tools for neo-epitope prediction have been described, including EpiToolKit, Epi-Seq, pVAC-Seq, and MuPeXI.^9–12^ These tools leverage tumor-sequencing, mass spectrometry, and epitope-predicting algorithms to identify mutant peptides and analyze structural features relevant to T cell receptor recognition, aiding the prediction of antigen immunogenicity.^6,8–12^ This is an imperative step to the development of a maximally effective cancer vaccine.

## Vaccine Formulation and Subtypes

A cancer vaccine combines the antigen component with an immune-stimulatory adjuvant. Antigens alone are poor inducers of the adaptive immune response. Stimulatory adjuvants enhance both innate and adaptive immunity by recruiting immune cells, promoting antigen trafficking to draining lymph nodes, and activating antigen-presenting cells (APCs) to initiate robust adaptive immune responses. These adjuvants can include agents such as toll-like receptor (TLR) agonists, cytokines and interferons, granulocyte macrophage colony-stimulating factor (GM-CSF), or targeted STimulator of INterferon Gene (STING) pathway modulators.

Depending on the formulation of the antigen component, vaccines can be classified into subtypes including cell-based vaccines and recombinant viral vaccines, as well as peptide-based and nucleic acid-based vaccines, each designed to optimize immune activation and tumor targeting. A selection of completed vaccine trials for immunologically cold solid tumors is described in Table 1.

**Table 1.** Completed perioperative vaccine trials in immunologically cold solid tumors

Year	Identifier	Phase	Cancer	Vaccine	Antigen	Treatment Design	Pt #	Primary outcome	Secondary outcomes
2013^13^	NCT00569387	II	Resectable PDAC	HAPa	Cell-based	(A) HAPa + Gem + 5FU	70	Positive: 12-month DFS 62%	12-month OS 86% Well tolerated
2016^16^	NCT01072981	III	Resectable PDAC	HAPa	Cell-based	(A) HAPa + SOC chemotherapy	722	Negative: mOS: 27.3 mo	3-yr OS 42%
						(A) SOC chemo		mOS: 30.4 mo	3-yr OS 41%
2022^17^	NCT01836432	III	BR-PDAC LAPC	HAPa	Cell-based	(NA) HAPa + SOC chemo and RT	303	Negative: mOS: 14.3 mo	mPFS 12.4 mo Grade 3 AE 81%
						(NA) SOC chemo and RT		mOS: 14.9 mo	mPFS 13.4 mo Grade 3 AE 75%
2011^20^	NCT00084383	II	Resected PDAC	GVAX	Cell-based	Adjuvant GVAX + 5FU + RT	60	Positive: DFS: 17.3 mo	mOS 24.8 mo Well tolerated Enhanced mesothelin-specific T-cell response
2021^21,22^	NCT00727441	II	Resectable PDAC	GVAX	Cell-based	(NA and A) GVAX+ SOC chemo	87	Negative: Peripheral level of mesothelin-specific T-cell response	mOS 35 mo TLA formation in 85% Higher TLA density correlated with longer OS
						(NA and A) GVAX+ 200mg Cy IV x 1+ SOC chemo			
						(NA and A) GVAX+ 100mg Cy PO x 1wk+ SOC chemo			
2023^23,24^	NCT02451982	II	Resectable PDAC	GVAX	Cell-based	(NA and A) GVAX+ Cy+ SOC chemo	16	Positive: Increased IL17A expression and intratumoral CD8+ CD137+ cells	mDFS 13.9 mo mOS 23.6 mo
						(NA and A) GVAX+ Cy + Anti-PD-1+ SOC chemo	14		mDFS 15.0 mo mOS 27.0 mo
						(NA and A) GVAX + Cy + Anti-PD-1+ CD137agonist + SOC chemo	10		mDFS 33.5 mo mOS 35.6 mo
2024^26^	NCT01595321	I	High-risk resected PDAC	GVAX	Cell-based	(A) GVAX+ Cy+ SBRT + SOC chemo	19	Positive: Safety	mOS 61 mo
2021^27^	NCT02648282	II	LAPC	GVAX	Cell-based	(NA) GVAX+ Cy+ SBRT+ Anti-PD-1 + SOC chemo	58	Negative: DFS <13.6 mo	Resected 44% Major pathologic response 42%
2023^28,29^	NCT03161379	II	BR-PDAC LAPC	GVAX	Cell-based	(NA) GVAX+ Cy+ SBRT+ SOC chemo	31	Positive: Increased CD8 T-cell density	mOS 20.4 months Major pathologic response 35%
2023^31^	NCT02779855	II/III	Stage 2-3 TNBC	TVEC	Viral-vector	(NA) TVEC+ SOC chemo	37	Positive: pCR: 46%	Major pathologic response 65% 2 yr DFS 89% Immune activation correlated with response
2007^41^	Not available	II	High-risk resected breast cancer	Theratope	Carbohydrate	(A) Theratope	27	Positive: Antibody response to STn	84% NED
2011^42^	Not available	III	Metastatic breast cancer	Theratope	Carbohydrate	Theratope + Cy + SOC chemo	1028	Negative: mOS, time to progression	Antibodies to STn Well tolerated
						Cy + SOC chemo			
2024^49^	NCT04853017	I	MRD PDAC and CRC	ELI-002	Pooled peptide	ELI-002	20 PDAC 5 CRC	Positive: Safety	KRAS-specific T-cell response 84% Tumor biomarker response 84% Tumor biomarker clearance 24%
2024^50^	NCT05726864	I/II	MRD PDAC	ELI-002 7P	Pooled peptide-antigen	ELI-002 7P	135	Awaiting report: Safety DFS	Awaiting report on T-cell response and tumor biomarkers
						Observation			
2023^52^	NCT04161755	I	Resected PDAC	Autogene cevumeran	mRNA	(A) autogene cevumeran + Anti-PD-L1 + SOC chemo	16	Positive: Safety	DFS not met vs 13.4 months in vaccine responders vs non responders, respectively
2024^55^	NCT04251117	I/II	Advanced HCC	GNOS-PV02	DNA	GNOS-PV02 + Anti-PD-1	36	Positive: Safety ORR: 31%	Complete response 8% neoantigen-specific T-cell response 86% mOS 30.2 v 15.7 mo stratified by neoantigen T-cell response

A = adjuvant; NA = neoadjuvant; Pt = patient; PDAC = Pancreatic Ductal Adenocarcinoma; HAPa = HyperAcute-Pancreas Algenpantucel-L; Gem = Gemcitabine; 5FU = 5-fluorouracil; DFS = Disease free survival; OS = overall survival; mOS = median overall survival; BR-PDAC = borderline resectable pancreatic ductal adenocarcinoma; LAPC = locally advanced pancreatic cancer; SOC = standard of care; mo = month; yr = year; RT = radiation therapy; AE = adverse events; GVAX = GM-CSF secreting allogenic PDAC vaccine; Cy = cyclophosphamide; TLA = tertiary lymphoid aggregates; SBRT = stereotactic body radiation therapy; TNBC = triple negative breast cancer; TVEC = talimogene laherparepvec; pCR = pathologic complete response; Theratope = Sialyly-Tn (STn) keyhole limpet hemocyanin vaccine; MRD = minimal residual disease, positive biomarkers despite no sign of recurrence on CT; CRC = colorectal cancer; ELI-002 = amphiphilic conjugated mutant KRAS G12D + G12R peptide vaccine; ELI-002 7P = amphiphilic conjugated mutant KRAS G12V, G12A, G12C, G12S, G13D, G12D + G12R peptide vaccine; HCC = hepatocellular carcinoma; GNOS-PV02 = Personalized DNA plasmid vaccine with plasmid-encoded IL12

## Cell-Based Vaccines

The antigenic component of cell-based vaccines is whole cancer cells, thus delivering a wide range of tumor antigens and circumventing the need to identify specific epitopes. With allogeneic vaccines, the anticipation is that a subset of antigens will be shared between the donor’s cells and the recipent’s cancer cells. This platform allows “off-the-shelf” availability, decreasing cost and increasing feasibility, but lacks personalization. A key challenge is tumor heterogeneity because emerging mutations may allow immune evasion, potentially limiting the efficacy of universal, cell-based vaccines.

### HyperAcute-Pancreas Algenpantucel-L (HAPa)

The HAPa is an allogeneic cancer vaccine formulation of two irradiated human PDAC lines modified to express murine α-1,3-galactosyltransferase. The ﻿α-galactosyl (﻿αGal) residues on their cell surface are targeted by naturally occurring anti-﻿α-Gal antibodies in humans, triggering complement-mediated hyperacute rejection and facilitating antibody-dependent, cell-mediated antitumor responses.^13–15^

A phase 2 trial (NCT00569387) investigated the impact of adding HAPa to gemcitabine or 5-fluorouracil (5FU) adjuvant chemotherapy for 70 patients with PDAC who had undergone R0 or R1 resection. The treatment was well-tolerated, with a 12-month disease-free survival (DFS) rate of 62 %, favorable in comparison with historic data.^13^

A subsequent, multi-institutional phase 3 IMPRESS trial (NCT01072981) investigated adjuvant HAPa in combination with standard-of-care (SOC) chemotherapy versus chemotherapy alone for 722 patients with resected PDAC. A median overall survival (OS) of 27.3 months was observed in the HAPa-combination group versus 30.4 months in the SOC chemotherapy group.^16^

The PILLAR trial was an additional phase 3 randomized trial (NCT01836432) assessing the efficacy and safety of HAPa in the neoadjuvant setting combined with SOC chemotherapy versus SOC chemoradiation therapy alone for patients with borderline resectable (BRPDAC) or locally advanced PDAC (LAPC).^17^ After successful randomization of 303 patients, the primary outcome of OS was not met during a median of 14.3 months in the experimental group and 14.9 months in the standard group.^17^ Additionally, there were no noted differences in DFS or survival stratified by baseline disease status. In light of the results from the PILLAR and IMPRESS trials, HAPa is no longer under consideration as a therapeutic option for PDAC.

### GM-CSF-Secreting Allogenic PDAC Vaccine (GVAX)

The GVAX is an irradiated GM-CSF-secreting allogeneic whole-cell PDAC vaccine shown to be safe in the advanced metastatic setting and associated with T cell-mediated systemic anti-tumor immunity.^18,19^ A phase 2 study (NCT00084383) investigated GVAX in 60 patients with resected PDAC and reported a median OS of 25 months. Although this did not differ significantly from the OS for matched historical control subjects, a significantly enhanced mesothelin-specific response was appreciated in the participants remaining disease-free longer than 3 years.^20^

This signal of immunogenicity in a previously cold tumor led to the investigation of GVAX in a neoadjuvant setting. Patients with resectable PDAC were enrolled in a phase 2 trial (NCT00727441) of neoadjuvant and adjuvant GVAX.^21^ Among the first 39 evaluable patients, organized tertiary lymphoid aggregates (TLAs) were identified in 85 % of tumors, compared with 0 % in the non-vaccinated historical control subjects.^20,21^ The median OS for the patients with neoadjuvant and adjuvant GVAX was 35 months compared with 24.8 months for historic control subjects treated with adjuvant GVAX alone.^20,22^ A higher density of TLA was associated with longer OS.^22^ The TLAs were associated with early T cell activation and a corresponding upregulation of immunosuppressive regulatory mechanisms within the PDAC TME through adaptive resistance such as PD-L1.^21^

The observed priming and associated upregulation of immunosuppressive pathways in the PDAC TME led to a logical next step of combining neoadjuvant GVAX with immune checkpoint inhibitors and immune costimulators. A phase 2 platform trial (NCT02451982) randomized patients with resectable PDAC to receive GVAX alone, GVAX plus anti-PD-1 antibody, or GVAX with both anti-PD-1 and anti-CD137.^23,24^ The primary immunologic end point of increased IL17A expression in vaccine-induced lymphoid aggregates was met in the GVAX versus GVAX + anti-PD-1 arms.^24^ Additionally, the primary end point of treatment-related changes in intratumoral CD8+CD137+ cells was met in the GVAX + anti-PD-1 arm versus the triplet combination arm (GVAX + anti-PD-1 + anti-CD137).^23,24^ The median survival outcomes were numerically favorable, but not statistically different between GVAX alone (DFS, 13.9 months; OS, 23.59 months) and combination GVAX, anti-PD-1, and anti-CD137 (DFS, 33.5 months; OS, 35.6 months).^23^

Pre-treatment tumor biopsy cores and post-treatment resected tumors were collected from 19 evaluable enrolled patients treated with GVAX or GVAX and anti-PD-1. Tertiary lymphoid aggregates were not appreciated in any of the pre-treatment core biopsies but were identified in all 19 tumor specimens after GVAX treatment with or without anti-PD-1.^24^ The addition of anti-PD-1 therapy to GVAX further increased the density of tumor-infiltrating CD4^+^ and CD8^+^ T cells while decreasing CD4^+^PD1^+^T cells compared with GVAX alone. However, merely increasing the number of effector T cells may not sufficiently translate to a survival advantage.^23,24^ Further optimization of effector T cell quality and activation is needed to truly optimize PDAC response to immunotherapy.^25^

In addition, GVAX has been studied in combination with other treatment methods. A phase 1 trial (NCT01595321) evaluated adjuvant GVAX in combination with stereotactic body radiation therapy (SBRT) and 5FU, irinotecan, oxaliplatin (FOLFIRINOX) chemotherapy for patients who had resected PDAC with high-risk features such as an R1 margin or positive lymph node metastases.^26^ The safety end points for this trial were met, with favorable exploratory survival outcomes during a median OS of 61 months for the patients receiving adjuvant GVAX and SBRT.^26^ Since the opening of this trial, there has been a growing preference for systemic neoadjuvant therapy to potentially reduce the risk of postoperative complications that might delay or prevent adjuvant chemotherapy.

Later studies explored the use of GVAX and SBRT as neoadjuvant therapy. The primary end point of DFS was not met in a phase 2 study (NCT02648282) of neoadjuvant SOC chemotherapy, GVAX, anti-PD1, and SBRT for patients with LAPC.^27^ A phase 2 trial (NCT03161379) of neoadjuvant FOLFIRINOX, SBRT, and GVAX again underscored the safety of this treatment combination during a median OS of 20.4 months, with a 35 % major pathologic response rate, comparable with contemporary studies.^28^ Correlative immunologic studies of treated resected tissue suggest that the addition of GVAX to SBRT increases densities of effector CD8^+^ T cells, with shorter distance to tumor cells. However, adaptive resistance was appreciated, with corresponding increases in immunosuppressive tumor-associated macrophages.^29^

Altogether, the many high-quality trials leveraging GVAX demonstrate the feasibility of investigating novel immunotherapy combinations through neoadjuvant platform trials for patients with immunologically cold tumors. Moreover, efficient multi-omic pre- and post-treatment tissue analyses permit an understanding of therapeutic resistance and response mechanisms and identify future targets for new therapeutic combinations.

## Recombinant Viral Vaccines

Oncolytic viral vaccines selectively infect and destroy tumor cells, releasing tumor antigens that stimulate an immune response. These viral vectors also can be engineered to express tumor-associated or other target antigens in infected tumor cells, which then present them as intracellular antigens. Additionally, because the viruses themselves are recognized as foreign, they trigger both innate and adaptive immune responses. However, a major limitation of recombinant viral vaccines is the development of host immunity against the viral vector, which can lead to its neutralization upon repeated administration, reducing efficacy.

### Talimogene Laherparepvec (TVEC)

The current most successful example of the viral vaccine platform is TVEC, an attenuated herpes simplex virus genetically modified to express GM-CSF. Durable response rates in advanced melanoma have led to Food and Drug Administration (FDA) approval of TVEC for regional disease.^30^ In addition, TVEC is being explored for other cancer subtypes, including its use in combination with neoadjuvant SOC chemotherapy for patients with stage 2 or 3 TNBC (NCT02779855).^31,32^ A pathologic complete response to combination treatment was observed in 46 % (*n* = 16) of patients, meeting the primary efficacy end point and supporting the safety of TVEC with chemotherapy.^32^ This study in TNBC supports further investigation of TVEC combined with chemotherapy in the neoadjuvant setting for immunologically cold tumors. Other oncolytic viral vaccine platforms, including the attenuated vaccinia virus Pexa-vec or modified adenovirus OBP-301, have been investigated in advanced HCC, with limited tumor responses warranting further investigation.^33–35^

## Peptide/Carbohydrate Antigen-Based Vaccines

The key to generating a robust immune response with peptide or carbohydrate vaccines is the combination of antigen and an immunostimulatory adjuvant. An immunologic adjuvant is a substance that acts to accelerate or enhance generated antigen specific immune responses. Peptides or carbohydrates alone are weakly immunogenic and may be limited by specific human leukocyte antigen (HLA) types, limiting applicability across diverse patient populations. Also, similar to limitations of cell-based approaches, the emergent predominance of certain mutations or downregulation of antigen expression can reduce vaccine efficacy over time.

### Sialyly-Tn and Keyhole Limpet Hemocyanin (KLH)

*Sialyly-Tn* (STn), a carbohydrate epitope found on a variety of glycoproteins, is associated with a poor prognosis for breast cancer patients.^36,37^ Synthetic STn combined with the KLH adjuvant (Theratope) demonstrated safety and ability to elicit an immune response in breast cancer patients.^38–40^ A phase 2 trial enrolled patients who had a history of breast cancer with no evidence of disease (NED) but at high risk due to increasing tumor markers or initial advanced disease stage. Significant antibody titers against STn developed in all the patients, and although the trial was not designed to assess clinical benefit, 84 % of the patients remained NED.^41^ However, a phase 3 clinical trial of Theratope involving 1028 women with metastatic breast cancer showed no improvement in time to progression or overall survival (OS) despite generation of strong antibody responses.^42^ This lack of efficacy may have been due to the advanced stage of metastatic disease or the limited therapeutic impact of the targeted antigen. This trial underscored the importance of identifying the right antigen, disease state, and understanding mechanisms of resistance in order to translate immunologic end points into survival.

### Amphiphile-Conjugated Mutant KRAS Peptide Vaccine (ELI-002)

The KRAS proto-oncogene plays a key role in tumor cell growth, proliferation, survival, and invasion, with mutations detected in more than 90 % of PDAC.^43–45^ Thus, mutant KRAS antigens are promising immunotherapy targets for many malignancies.^46^ The ELI-002 vaccine combines lipid-conjugated mutant KRAS-derived peptides, G12D, and G12R with a lipid-conjugated immune-stimulatory oligonucleotide. Amphiphilic technology harnesses the lipid component’s ability to bind to albumin and deliver the specific antigen payload to lymph nodes for specific T cell priming and anti-tumor efficacy.^47,48^

The AMPLIFY-201 phase 1 study (NCT04853017) investigated ELI-002 in patients with KRAS-mutated pancreatic and colorectal cancer who had received curative-intent resection and adjuvant therapy but were positive for minimal residual disease (MRD).^49^ The therapy was well-tolerated without dose-limiting toxicity, and a KRAS-specific T cell response was observed in 21 of 25 patients, with an average 58-fold increase from baseline.^49^ Biomarkers of circulating tumor (ct) DNA and/or cancer antigen 19-9 (CA19-9) were reduced from baseline in 84 % of the patients, with complete clearance in 24 % of patients. Efficacy correlated with T cell responses, with significantly improved DFS for patients exceeding the median T cell response.^49^

The next-generation vaccine, ELI-002 7P, targets G12V, G12A, G12C, G12S, and G13D mutations in addition to G12D and G12R, accounting for 90 % of pancreatic cancer patients. The AMPLIFY-7P (NCT05726864) trial evaluated multiple antigen doses in a phase 1 study followed by a preplanned phase 2 study enrolling an additional 135 PDAC patients randomized 2:1 to adjuvant ELI-0027P or observation. Use of ELI-002 7P was safe, with the median recommended phase 2 antigen dose T cell responses exceeding those of the first-generation vaccine.^50^ The phase 2 study has recently completed recruitment.

## Nucleic Acid-Based Vaccines

Nucleic acids can be transfected into somatic cells to express a target antigen. For transcription and translation, DNA plasmids encoding T cell epitopes must enter the nucleus, but do not integrate into the host genome. In contrast, RNA requires only cytoplasmic entry for translation and avoids genomic integration, although effective delivery systems are needed to prevent degradation. Lipid nanoparticles represent a major breakthrough for RNA therapeutics, enabling their successful clinical delivery. This was most notably showcased through the effective delivery of the COVID-19 vaccines, offering potential for broader therapeutic applications.^51^

### Lipoplex Nanoparticle RNA Neoantigen Vaccine (Autogene Cevumeran)

Autogene cevumeran is an individualized therapeutic RNA vaccine containing up to 20 personalized neoantigens encapsulated in a lipid nanoparticle delivery system. The patient-specific neoantigens are identified using bioinformatic discovery informed by genomic and transcriptomic data from resected tumor specimen.

A phase 1 study (NCT04161755) investigated the safety and immunogenicity of adjuvant autogene cevumeran and anti-PD-L1 therapy in combination with SOC chemotherapy for 16 patients with resected PDAC.^52^ The primary end point of safety was met. During a median follow-up period of 18 months, the patients with vaccine-induced T cell responses to at least one neoantigen had a longer median DFS (median RFS not reached) than the patients without an immunologic response (median RFS, 13.4 months).^52^ Subsequent analyses demonstrated that the vaccine-induced T cells were durable, with 86 % of vaccine-induced T cell clones persisting approximately 3.8 years after vaccination. Importantly, the vaccine response continued to correlate with recurrence-free survival (RFS) during a 3-year median follow-up period (median RFS not reached for responders vs 13.4 months for non-responders; hazard ratio [HR], 0.14; 95 % confidence interval [CI], 0.03–0.59).^53^ The phase 2 IMCODE003 clinical trial (NCT05968326) is ongoing to evaluate the efficacy of autogene cevumeran plus anti-PD-L1 therapy and SOC chemotherapy for participants with resected PDAC versus adjuvant SOC chemotherapy.

### Personalized DNA Plasmid Vaccine With Plasmid-Encoded IL12 (GNOS-PV02)

The plasmid-based vaccine, GNOS-PV02, encodes up to 40 personalized neoantigens, identified from each patient's tumor through exome and transcriptome sequencing. A second plasmid expresses interleukin 12 (IL12), which acts as the adjuvant.^54,55^ In a single-arm, open-label phase 1/2 study (NCT04251117), GNOS-PV02 was studied in combination with anti-PD-1 for patients who had advanced HCC previously treated with multityrosine kinase inhibitors.^55^ The treatment was safe and well-tolerated in all 36 patients, with a 31% objective response rate, including an 8 % complete response rate. The patients in the top 25% of the neoantigen-specific T cell responses showed a trend toward longer OS than those in the bottom 25% (30.2 vs 15.7 months).^55^ Although this study treated an advanced, refractory HCC population, one unresectable patient achieved secondary resectability after tumor shrinkage, with an 18-month disease-free interval after treatment.^55^ This conversion to resectability highlights the potential for further investigation of this treatment platform as an induction therapy for locally advanced disease.

## Future of Cancer Vaccines

Whereas novel immunotherapeutic platforms are most commonly investigated in the advanced disease setting, the tumor microenvironment (TME) in metastatic cancer differs significantly from that of the primary tumor, presenting challenges in tracking immune responses and resistance mechanisms over time.^56^ The neoadjuvant and window-of-opportunity approaches offer a distinct advantage because the intact primary tumor provides antigen to elicit immune responses, with subsequent resection enabling robust assessments of the TME and key pathways influencing the anti-tumor response.^21,56^

A deeper understanding of surrogate outcomes is needed for accurate evaluation of cancer vaccine responses. Cancer vaccines exhibit delayed and progressive immune activation, unlike cytotoxic drugs, which induce immediate tumor regression through direct tumor killing. Therefore, conventional end points such as progression-free survival (PFS) may be inadequate for evaluating vaccine efficacy, highlighting the need for immune-based surrogate outcomes to better assess long-term therapeutic impact. For example, comparisons of pre- and post-treatment tissue of immune cell infiltration and T cell repertoire expansion may better capture vaccine-induced immune memory. These outcome measures and the future development of immune response biomarkers are easiest to accomplish with pre- and post-treated tissue available in the window-of-opportunity setting.

Interest is growing for rational combinatorial regimens with a vaccine backbone that target separate steps of anti-tumor immunity.^57^ This potential synergistic effect includes other immunotherapy agents that may not be efficacious in isolation.^58–60^ In contrast to checkpoint inhibitors, which reinvigorate preexisting anti-tumor immunity, cancer vaccines prime immunologically cold tumors for *de novo* immune responses (Fig. 1). As safety and preliminary efficacy are established, studying cancer vaccines in combination with other immunotherapeutics in the neoadjuvant setting may offer the best opportunity to enhance our understanding of T cell-specific anti-tumor immune response underlying mechanisms that drive therapeutic resistance and potentially expand the armamentarium for a host of immunologically cold tumors with limited treatment options.

The immunosuppressive tumor microenvironment in cold tumors limits immune responses through low neoantigen availability, defective antigen presentation, immunosuppressive antigen-presenting cells (APCs), dense stroma blocking infiltration, and inhibitory signaling that prevents T cell recruitment and activation. Additionally, dysregulated chemokines favor suppressive cell infiltration, whereas exhausted effector cells and suppressive immune populations further restrict anti-tumor immunity. A multi-pronged immunotherapy approach can overcome these barriers at different stages of the immune response. Radiotherapy, oncolytic viral therapy, and cancer vaccines increase tumor-specific antigen availability, whereas cytokines and cGAS-STING agonists enhance antigen processing and APC activation. Stromal modulators, T cell agonists, CAR-T cells, and immune checkpoint blockade improve T cell-trafficking and infiltration, enabling more robust immune engagement. Finally, checkpoint inhibitors and therapies targeting immunosuppressive pathways enhance the function of infiltrating effector cells, maximizing anti-tumor activity.
